# Enzymatic Pea Protein Hydrolysates Are Active Trypsin and Chymotrypsin Inhibitors

**DOI:** 10.3390/foods8060200

**Published:** 2019-06-10

**Authors:** Temitola Awosika, Rotimi E. Aluko

**Affiliations:** 1 Department of Food and Human Nutritional Sciences, University of Manitoba, Winnipeg MB R3T 2N2, Canada; awosikat@myumanitoba.ca; 2 Richardson Centre for Functional Foods and Nutraceuticals, University of Manitoba, Winnipeg, MB R3T 2N2, Canada

**Keywords:** pea, protein hydrolysate, trypsin, chymotrypsin, enzyme inhibition kinetics, protease inhibitors

## Abstract

In this work, we report the potency of enzymatic hydrolysates of pea proteins against trypsin and chymotrypsin. Pea protein concentrate was digested with each of alcalase, chymotrypsin, pepsin, and trypsin, followed by membrane separation of the protein hydrolysates into peptide fractions (<1, 1–3, 3–5, and 5–10 kDa). Peptide size profiling with size-exclusion gel chromatography indicated the narrowest size range (0.85–4.98 kDa) for alcalase. Trypsin activity was strongly (*p* < 0.05) inhibited by the ultrafiltration fractions (mean IC_50_ = 2.2 mg/mL) obtained from the trypsin hydrolysate when compared to the unfractionated hydrolysate (IC_50_ = 6.8 mg/mL). Similarly, ultrafiltration also enhanced trypsin inhibition by the alcalase-digested peptides with an IC_50_ of 21.4 mg/mL for the unfractionated hydrolysate in comparison to 3.1–4.7 mg/mL for the fractions. However, ultrafiltration did not enhance trypsin inhibitory activity of chymotrypsin-digested peptides, while the peptide separation reduced efficacy of pepsin-digested peptides. In contrast, chymotrypsin inhibition by all the enzymatic digests was significantly (*p* < 0.05) enhanced by ultrafiltration, especially peptide sizes >3 kDa. Kinetics of enzyme inhibition indicate peptides were bound to the enzyme active site in a competitive mode that led to reduced catalysis. We conclude that the pea peptides could function as useful tools to promote human health and as a preservative during food processing and storage.

## 1. Introduction

Food-derived protease inhibitors have essentially been in the form of large proteins that block activities of trypsin and chymotrypsin, the major serine proteases. The initial serine protease inhibitors were isolated from soybean seeds as the Kunitz inhibitor, which predominantly acts against trypsin [1], while the Bowman–Birk inhibitor (BBI) is effective against trypsin and chymotrypsin [2]. Subsequently, protease inhibitors have been isolated from other plant [3,4,5] and animal [6,7,8,9] tissues, as well as microbes [10,11]. The physiological roles of protease inhibitors are diverse and include regulation of various cellular activities, prevention of organ structure deterioration (e.g., the pancreas), and inhibition of viral infections [12]. This is because proteases are involved in the irreversible hydrolysis of peptide bonds; therefore, unregulated proteolysis can lead to serious metabolic disorders that precipitate the onset and progression of diseases such as cancer, cardiovascular disorders, neurodegeneration, and chronic inflammation [12,13,14,15,16,17]. As a preventive tool, protease inhibitors have been shown to inhibit proliferation of preadipocytes, which suggests an anti-obesity potential [18]. Serine protease inhibitors have also been associated with ability to potentiate the immune system and blood development [15,19,20]. In addition to their physiological roles, protease inhibitors also serve as food processing agents that can be used to prevent or limit the extent of unwanted proteolytic degradation in animal muscles and cereal flours. For example, proteolytic degradation of gluten will reduce bread loaf volume and product quality. However, incorporation of natural protease inhibitors into such high protease flours can assist in preventing deterioration of gluten quality and enable production of high quality dough [21]. Similarly, the textural properties of meat and fish products have been shown to be dependent on the activity level of protease inhibitors [22,23,24,25,26]. In terms of grain preservation, the presence of protease inhibitors in seeds have been shown to limit insect infestation during storage [27]. 

However, inhibition of the enzymes (trypsin and chymotrypsin) involved in protein digestion is also paramount as it is known that excessive protein intake can cause weight gain [28] as excess amino acids are used to synthesize acetyl-CoA, a precursor for the synthesis of triglycerides, which in turn is stored as fat in the body. The excess amino acids can also be used as substrates for the production of glucose during gluconeogenesis [29]. In addition, a high protein diet can stress or overwork the kidney as increased breakdown of proteins produce high levels of ammonia or urea, which are waste and toxic products that are excreted through the kidney [30]. Synthetic digestive enzyme inhibitors have been developed for various uses; however, they tend to possess negative side effects ranging from diarrhea to hepatotoxicity [31]. With the global prevalence of obesity and type-2 diabetes mellitus (T2DM), identification of alternative enzyme inhibitors becomes imperative. Compounds of natural sources (such as dietary components) are more desirable as they are thought to possess lower risk of negative side effects when compared to the synthetic inhibitors [32].

In contrast to whole proteins, food-derived peptides are usually obtained from the enzymatic hydrolysis of animal and plant proteins [33]. Depending on the source, type of enzyme, and processing conditions, these peptides could influence several metabolic processes to impart health benefits. For example, food protein-derived peptides have been proven to have various physiological effects that include free radical scavenging, blood pressure reduction, immunomodulation, bone development, neuroprotection, and renoprotection [34]. However, with the exception of two recent works showing the inhibition of BBI by a cyclic nonapeptide [35] and 4 kDa seed peptides [36], information is scant regarding serine protease inhibition by low molecular weight peptides, especially from enzymatic digests of food proteins. Therefore, we hypothesized in this study that peptides with inhibitory activities against trypsin and chymotrypsin can be isolated after hydrolysis of pea protein with alcalase, pepsin, trypsin, and chymotrypsin. Since products of an enzyme catalytic reaction can act as feedback inhibitors of the same enzyme, digestive enzymes were used to enhance the possibility of producing potent peptide inhibitors against the respective catalyst (pepsin, trypsin, and chymotrypsin). Enzyme kinetic studies were also conducted to determine the possible mode of inhibition by the peptides. 

## 2. Materials and Methods 

### 2.1. Materials

The yellow field pea protein concentrate (PPC) containing 82% protein content (dry wt basis) was purchased from Nutri-Pea Limited (Portage la Prairie, MB, Canada). BApNA (N-Benzoyl-D-L arginine *p*-nitroanilide), BTpNA (*N*-Benzoyl-l-tyrosine *p*-nitroanilide), 4-(2-Aminoethyl) benzenesulfonyl fluoride hydrochloride (AEBSF), bovine chymotrypsin, porcine pancreatic trypsin, and other analytical grade reagents were purchased from Sigma-Aldrich (St. Louis, MO, USA) and Fisher Scientific Company (Oakville, ON, Canada), respectively.

### 2.2. Preparation of Yellow Field Pea Protein Hydrolysates

PPC was hydrolyzed (in duplicates) using four different proteases (alcalase, pepsin, trypsin, and chymotrypsin) as previously described [37]. The enzyme hydrolysis conditions are shown in Table 1. Briefly, the PPC (5%, *w/v*) was suspended in double distilled water followed by heating to optimal temperature. After the protein mixtures have been adjusted to the optimal pH of each protease, hydrolysis was initiated by addition of each protease at a ratio of 1:25 enzyme/substrate ratio. Hydrolysis was allowed to proceed for 4 h accompanied by constant adjustment to the set pH value using a 0.5 M NaOH solution for alcalase, trypsin, and chymotrypsin reactions while 1 M HCl was used for the pepsin reaction. The duration of hydrolysis was based on lack of change in pH of the reaction mixture after 4 h, which indicates no further hydrolysis occurred beyond this point. Hydrolysis was then terminated by adjusting to pH 5.0 and the mixtures heated at 95 °C for 15 min to completely inactivate the enzymes. The digestion mixture was cooled to room temperature and then centrifuged (10,000× *g* for 15 min at 4 °C). The supernatant was collected, and a portion freeze-dried and stored at −20 °C as the protein hydrolysate. The remaining portion of the supernatant was separated into different peptide fractions using membrane ultrafiltration.

### 2.3. Size-Exclusion Column Chromatography Analysis of Protein Hydrolysates 

Determination of the peptide size distribution of the four pea protein enzymatic hydrolysates was carried out using the method of Alashi et al. [38]. In summary, the hydrolysates (10–15 mg/mL) were dissolved in 0.05 M sodium phosphate buffer containing 0.15 M NaCl (pH 7.2) and then filtered through a 0.2 μm syringe filter. The filtrates were then injected (1 mL) onto a Superdex Peptide 10/300 (10 × 300 mm) GL column attached to an Fast Protein Liquid Chromatography system (AKTA purifier, GE Healthcare, Montreal, PQ, Canada) equipped with a UV detector. Isocratic peptide elution was carried out with the phosphate buffer using 0.5 mL/min flow rate and elution monitored at 214 nm. The column was calibrated with cytochrome C (12.384 kDa), aprotinin (6.512 kDa), vitamin B12 (1.855 kDa), and glycine (0.075 kDa) as molecular weight (MW) standards. Peptide sizes in the protein hydrolysates were estimated from a plot of log MW versus elution volume of the standards.

### 2.4. Preparation of Yellow Field Pea Protein Peptide Fractions via Ultrafiltration

The membrane ultrafiltration protocols were carried out according to the method of Awosika and Aluko [37]. Briefly, the supernatant obtained after enzyme digestion was fractionated into different peptide sizes in a sequential manner using an Amicon stirred ultrafiltration system (Millipore Sigma, Merck KGaA, Darmstadt, Germany) fitted with 1, 3, 5, or 10 kDa MW cut-off membrane. First, the supernatant was filtered through the 1 kDa membrane and the permeate (<1 kDa) collected, while the retentate was mixed with an equal amount of water and then filtered through a 3 kDa membrane to obtain a 1–3 kDa permeate. The filtration protocol was repeated by filtering the diluted 3 kDa retentate through a 5 kDa membrane to collect a permeate (3–5 kDa), while the 5 kDa retentate was passed through a 10 kDa membrane and the permeate collected as the 5–10 kDa fraction. The permeates were freeze-dried and stored at −20 °C. The freeze-dried protein hydrolysates and ultrafiltration fractions were analyzed for protein content using the modified Lowry method [39]. 

### 2.5. Inhibition of Trypsin Activity

Trypsin inhibitory activity was determined following a previously described method [40]. Briefly, 200 μL of trypsin (dissolved in 20 mM Tris-HCl buffer, pH 7.5; final concentration of 60 µg/mL) was premixed with 200 μL of samples (dissolved in the above buffer; final peptide concentration of 1–10 mg/mL) and incubated for 5 min at 37 °C. The reaction was started by the addition of 500 μL of 1 mM BApNA prepared in Tris HCl buffer (pH 7.5) that contained 1% (*v/v*) dimethyl sulfoxide. Following incubation for 10 min at 37 °C, the reaction was terminated by the addition of 100 μL of 30% (*v/v*) acetic acid. A 200 µL aliquot of the reaction mixture was transferred to a 96-well microplate and the absorbance measured using a microplate reader at 410 nm. Trypsin inhibitory activity was determined by measuring the release of *p*-nitroanaline from BApNA. AEBSF was used as a positive control. The trypsin inhibitory activity (%) was calculated using the equation:

Inhibition (%) = ((Ac − As)/Ac) × 100

where Ac = absorbance of the control; As = absorbance of the sample. The 50% inhibition concentration (IC_50_) values of the protein hydrolysates and membrane fractions were obtained by non-linear regression analysis of a plot of trypsin percentage inhibition versus the sample concentrations using GraphPad Prism version 6.0 (GraphPad Software, San Diego, CA, USA).

### 2.6. Inhibition of Chymotrypsin Activity

Chymotrypsin-inhibitory activity was determined using previously described protocols [27]. Briefly, 400 μL of chymotrypsin (dissolved in 0.01 M Tris-HCl buffer containing 0.02 M CaCl_2_, pH 8.0; final concentration = 20 µg/mL) and 400 μL of samples (re-dissolved in the same buffer; final peptide concentration of 2–6 mg/mL) were pre-mixed and incubated for 15 min at 37 °C. The reaction was started by the addition of 1 mL of 1 mM BTpNA prepared in 0.01 M Tris-HCL buffer containing 0.02 M CaCl_2_ and 40% (*v/v*) ethanol, pH 8.0. Following incubation for 15 min at 37 °C, the reaction was terminated by the addition of 200 μL of 30% (*v/v*) acetic acid. A 200 µL aliquot of the reaction mixture was transferred to a 96-well microplate and the absorbance measured using a microplate reader at 410 nm. Chymotrypsin inhibitory activity was determined by measuring the release of *p*-nitroanaline from the substrate BTpNA. AEBSF was used as a positive control. The chymotrypsin inhibitory activity (%) was calculated using the equation:

Inhibition (%) = ((Ac − As)/Ac) × 100

where Ac = absorbance of the control; As = absorbance of the sample. The IC_50_ values were determined as described above for trypsin.

### 2.7. Kinetics of Enzyme Inhibition

The mode of enzyme inhibition by protein hydrolysates and membrane fractions was determined for trypsin and chymotrypsin by conducting enzyme reactions at various substrate and peptide concentrations [27]. The inhibition pattern was then determined from the double reciprocal Lineweaver–Burk plots, in which the inverse of the initial rate was plotted against the inverse of the substrate concentration in the presence or absence of the peptides. BApNA in the range 0.5–2.5 mM and BTpNA in the range 0.05–0.3 mM were used as substrate concentrations for trypsin- and chymotrypsin-catalyzed reactions, respectively. The Lineweaver–Burk plots and kinetic parameters were obtained using GraphPad Prism version 6.0 (GraphPad Software, San Diego, CA, USA).

### 2.8. Statistical Analysis

All assays were conducted in triplicate and data analyzed as the mean values ± standard deviation. The mean values were examined using analysis of variance (ANOVA) and then compared using Duncan’s multiple range test with significant differences accepted at *p* < 0.05. All analyses were conducted using Statistical Package for the Social Science version 16.0 (IBM Corporation, Armonk, NY, USA).

## 3. Results

### 3.1. Peptide Size Distribution

**Figure 1:**
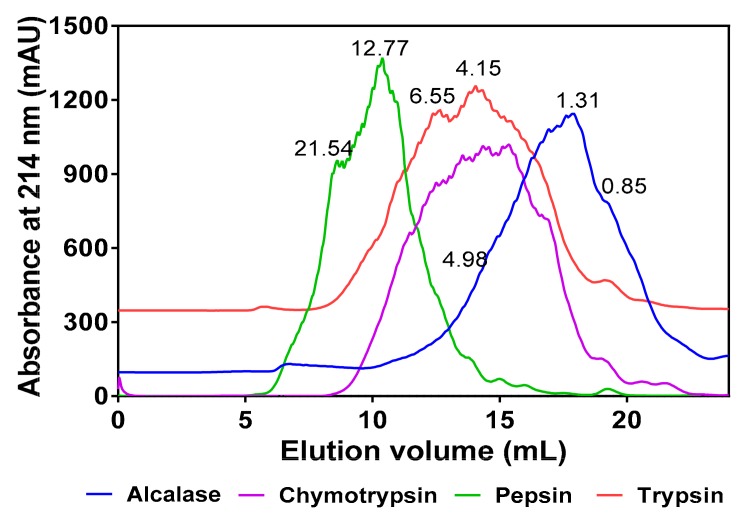
Comparative gel-permeation chromatograms of the four protein hydrolysates after passage through a Superdex Peptide12 10/300 GL column. Inserted values indicate estimated molecular weights (kDa).

As shown in Figure 1, the MW range of peptides present in the hydrolysates differed according to the protease used for digestion. Alcalase hydrolysate consisted of peptides within the 0.85–4.98 kDa size range, which is narrower than those of chymotrypsin (0.41–9.14 kDa), trypsin (0.85–13.57 kDa), and pepsin (0.88–21.54 kDa). Based on the late-eluting peak, the alcalase hydrolysate also consisted of more low MW peptides than the other three protein hydrolysates. The peptide size distribution was similar for chymotrypsin and trypsin hydrolysates while the pepsin hydrolysate contained a wider distribution of high MW peptides. 

### 3.2. Trypsin Inhibition

**Figure 2:**
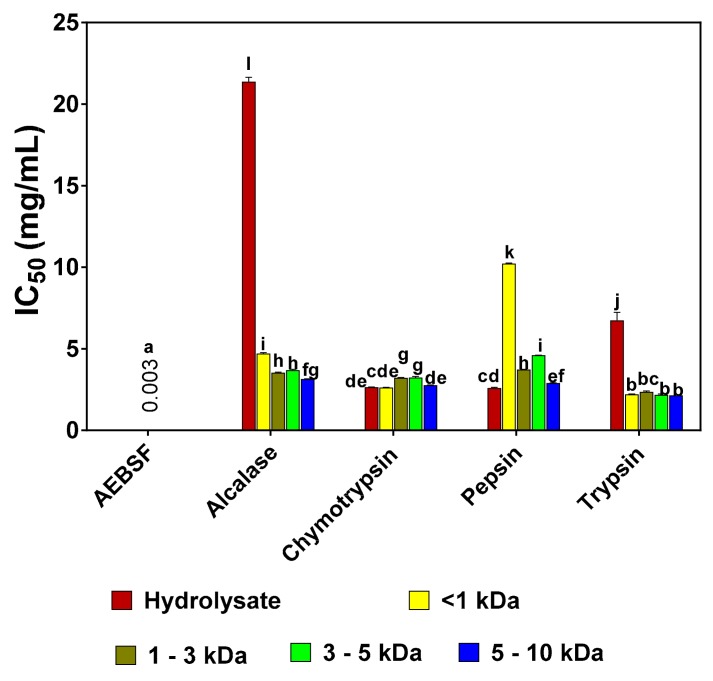
Inhibitory concentrations of 4-(2-Aminoethyl) benzenesulfonyl fluoride hydrochloride (AEBSF) in comparison to those of pea protein hydrolysates and peptide fractions that reduced trypsin activity by 50% (IC_50_). Results are presented as mean ± standard deviation. Bars with different letters have significantly different (*p* < 0.05) mean values.

Results of the IC_50_ values for the trypsin-inhibitory activity of pea protein hydrolysates and fractions are presented in Figure 2. Generally, the results showed that the peptide fractions had lower IC_50_ values when compared to the unfractionated hydrolysates, except for pepsin-derived hydrolysate, which had a lower IC_50_ in comparison to the other pepsin-derived peptide fractions. Overall, trypsin 5–10 kDa peptide fraction had the lowest IC_50_ value of 2.14 mg/mL. In the alcalase group, alcalase 5–10 kDa fraction had the lowest IC_50_ value of 3.14 mg/mL, whereas in the chymotrypsin group, the <1 kDa fraction had the lowest IC_50_ value of 2.63 mg/mL. However, in comparison to the standard trypsin inhibitor drug (AEBSF with an IC_50_ value of 0.003 mg/mL), the IC_50_ values of pea protein hydrolysates and peptide fractions were significantly (*p* < 0.05) higher.

### 3.3. Chymotrypsin Inhibition

**Figure 3:**
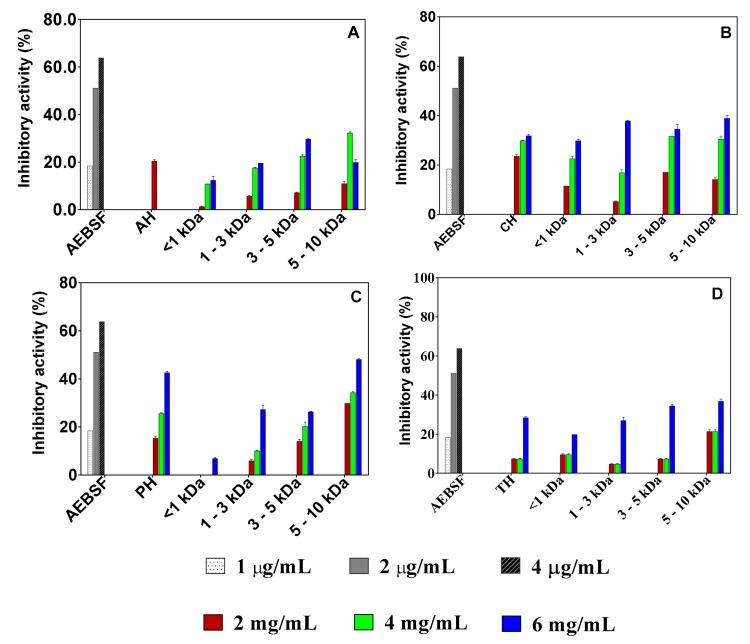
Concentration-dependent inhibition of chymotrypsin activity by 4-(2-Aminoethyl) benzenesulfonyl fluoride hydrochloride (AEBSF) in comparison to those of protein hydrolysates (AH, alcalase hydrolysate; CH, chymotrypsin hydrolysate; PH, pepsin hydrolysate; TH, trypsin hydrolysate) and peptide fractions obtained with alcalase (**A**), chymotrypsin (**B**), pepsin (**C**), and trypsin (**D**). Results are presented as mean ± standard deviation.

Percentage chymotrypsin inhibition by the pea protein hydrolysates and peptide fractions was concentration dependent in the 2–6 mg/mL range, as shown in Figure 3. IC_50_ values could not be determined because percent inhibition did not reach 50% even when sample concentrations exceeded 6 mg/mL. Inhibitory activities of the samples were lower than that of the standard AEBSF (66.39% at 6 μg/mL). Based on the results of the highest concentration tested (6 mg/mL), the pepsin 5–10 kDa fraction had the highest inhibitory activity of 48.13% among all the samples. With regards to each group, the alcalase 3–5 kDa peptide fraction had higher inhibitory activity (29.68%) than the other fractions. In contrast, the 5–10 kDa fraction was the most active within each group of trypsin (36.93 ± 1.04%) and chymotrypsin (38.90%).

### 3.4. Kinetics of Trypsin Inhibition

**Figure 4:**
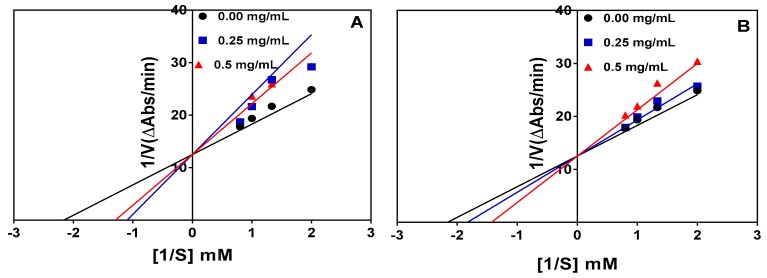
Lineweaver–Burk plots of trypsin inhibition at different peptide concentrations: (**A**) trypsin hydrolysate (*R*^2^ = 0.99, 0.89, and 0.87 for 0.0, 0.25, and 0.5 mg/mL, respectively) and (**B**) trypsin hydrolysate <1 kDa peptide fraction (*R*^2^ = 0.99, 0.98, and 0.96 for 0.0, 0.25, and 0.5 mg/mL, respectively).

The Lineweaver–Burk plots for the trypsin-catalyzed reaction in the absence and presence of the peptide inhibitors at two concentrations are shown in Figure 4A,B. The results indicate a competitive type of inhibition because the lines intersected at the y-axis at the same point. Therefore, in competitive inhibition, the inhibitors do not affect the Vmax of the enzyme (i.e., Vmax is unchanged) but increases the Km. The results of the kinetic parameters are shown in Table 2. The Vmax values for both the hydrolysate and <1 kDa peptide fraction remained unchanged (0.08) but the Km value was higher than that of the control (uninhibited) reaction. The inhibition constant (Ki), which indicates the binding ability to the trypsin enzyme, revealed that the <1 kDa peptide fraction (1.073 mg/mL) binds stronger than trypsin hydrolysate (1.172 mg/mL).

### 3.5. Kinetics of Chymotrypsin Inhibition

**Figure 5:**
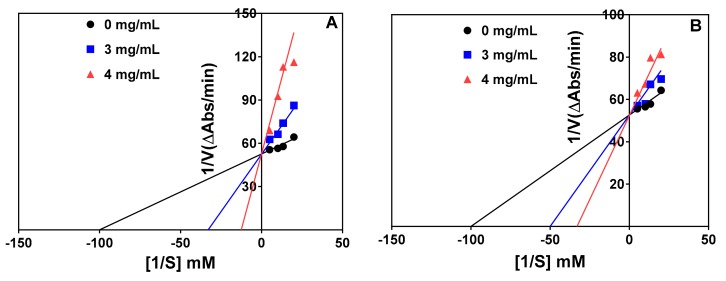
Lineweaver–Burk plots of chymotrypsin inhibition at different peptide concentrations: (**A**) chymotrypsin hydrolysate (*R*^2^ = 0.88, 0.96, and 0.85 for 0, 3, and 4 mg/mL, respectively) and (**B**) chymotrypsin hydrolysate 1–3 kDa peptide fraction (*R*^2^ = 0.88, 0.84, and 0.84 for 0, 3, and 4 mg/mL, respectively).

Inhibition of the chymotrypsin-catalyzed reaction was also competitive as shown by the intersection of the lines at the same point on the y-axis in Figure 5A and 5B for the protein hydrolysate and 1–3 kDa peptide fraction, respectively. Therefore, the Vmax remained unchanged whereas there were increases in the Km value in the presence of the protein hydrolysate and peptide fraction, as shown in Table 3. The protein hydrolysate had a lower Ki value (0.763 mg/mL), which indicates higher affinity than the 1–3 kDa peptide fraction (1.897 mg/mL) during interactions with the chymotrypsin enzyme. 

## 4. Discussion

Determination of the molecular weight of hydrolysate is a key criterion in considering the potential bioactivities of peptides. This is because low molecular weight peptides have better chances of escaping structural degradation within the gastrointestinal tract and being absorbed into blood circulation than larger peptides. In addition, small-sized peptides tend to fit and bind tightly into an enzyme’s active site and produce stronger inhibitory effects than larger peptides. As a result, one of the objectives of protein hydrolysate production is to ensure the abundance of small-sized peptides in order to enhance potency against metabolic targets [41]. The alcalase hydrolysate showed more low MW peaks, indicating greater proteolytic efficiency against the pea protein when compared to chymotrypsin, trypsin, and pepsin. The results are consistent with our previous work that reported a greater number (21) of di- and tripeptide sequences in the alcalase hydrolysate in comparison to only four in the chymotrypsin hydrolysate and none in the pepsin and trypsin hydrolysates [37]. The differences in peptide sizes between the hydrolysates can be attributed to variations in hydrolytic specificity of the enzymes. A previous report also reported that the structure (size) and activity of peptides are largely dependent on their method of production, i.e., type of enzyme used [42]. The results obtained in this study for the alcalase hydrolysate is in the 0.027–6.47 kDa range that was reported for canola protein hydrolysates [38]. Moreover, alcalase has been reported to produce smaller molecular weight peptides probably due to its broad specificity during protein digestion [41,43,44,45].

The enzyme inhibition results indicate that fractionation improved the trypsin- and chymotrypsin-inhibitory capacity of pea protein hydrolysates, depending on the hydrolytic enzyme. For example, alcalase and trypsin hydrolysates had weaker trypsin-inhibitory activity than the ultrafiltration fractions. Similarly, the >3 kDa fractions of all the hydrolysates had stronger chymotrypsin inhibition than the unfractionated hydrolysates. The results suggest that there may have been peptide antagonism within the unfractionated hydrolysates, which could have contributed to reduced trypsin and chymotrypsin-inhibitory activities. Ultrafiltration separation could have reduced antagonistic effects in the fractionated peptides, hence the higher inhibitory effects of the peptide fractions. Similar results were reported for cod protein hydrolysates and the ultrafiltration fractions [46]. The beneficial effect of ultrafiltration membrane separation was not observed for trypsin inhibition by the chymotrypsin and pepsin hydrolysates. The differences in the effect of ultrafiltration could be attributed to the presence of varied types (peptide sequences) and amounts of peptides within each hydrolysate as we have previously reported [37]. The varied response of the protease inhibitors to ultrafiltration fractions is consistent with the known catalytic differences between trypsin and chymotrypsin. This is because chymotrypsin activity is specific for peptide bonds where the carbonyl group is donated by aromatic amino acids, such as tyrosine, phenylalanine, and tryptophan, while trypsin catalyzes bonds with the carboxyl side donated by arginine and lysine [47]. A previous work also showed that peptides inhibited trypsin activity but not chymotrypsin [36]. Overall, the pea protein hydrolysates and peptide fractions are significantly (*p* < 0.05) weaker trypsin and chymotrypsin inhibitors when compared to AEBSF, which is considered a standard protease inhibitor. In comparison to this work, an isolated polypeptide from Chinese cabbage seeds inhibited trypsin with an IC_50_ value of 8.5 µM [48]. However, the pea peptides are stronger chymotrypsin inhibitors when compared to the two protease-inhibitory peptides extracted from *Opuntia joconostle* Weber seeds, which were inactive against this enzyme [36].

In this present study, the modes of inhibition of trypsin and chymotrypsin activities were investigated via kinetic studies in the absence and presence of the enzymatic hydrolysates or the chosen peptide fractions. Lineweaver–Burk plots were used to determine the possible mode of enzyme inhibition and the results indicate a competitive type of inhibition for both enzymes. Therefore, the peptides were bound to the active site of both enzymes, which reduced the rate of substrate binding, hence the lower catalytic activities than when the peptides were not present. Since increases in substrate concentration could overcome the initial inhibition by the peptides, the maximum velocity was the same for inhibited and non-inhibited reactions. However, binding of the peptides to the active site necessitated an increase in substrate concentration required to reach half-maximal velocity, hence the Km values were higher for the inhibited reactions. In other words, binding of the peptide samples affected Km of the reaction catalyzed by both enzymes, proportionately to peptide concentration without affecting maximum velocity (Vmax) of the reaction. The results are similar to our previous report, which also observed that the chymotrypsin pea protein hydrolysate inhibited α-amylase in a competitive manner; in contrast, inhibition of α-glucosidase was non-competitive [37]. Trypsin inhibition by a horse gram protease inhibitor was also shown to be competitive [49], which is consistent with the results obtained for the pea protein hydrolysates and peptide fractions. The inhibition constant (Ki) provides an indication of the binding affinity of inhibitors to an enzyme target; lower values suggest stronger binding affinity than higher values. Therefore, the <1 kDa peptide fraction has a slightly stronger affinity to trypsin when compared to the unfractionated trypsin hydrolysate. In contrast, the unfractionated chymotrypsin hydrolysate with a lower Ki value (0.763 mg/mL) had greater affinity for chymotrypsin protein when compared to the 1–3 kDa peptide fraction (1.897 mg/mL).

## 5. Conclusions

For the first time, we report the ability of enzymatic protein hydrolysates to reduce the catalytic rate of trypsin- and chymotrypsin-catalyzed reactions. Peptide size varied, with alcalase producing the smallest peptides, but this factor did not have any consistent effect on potency of enzyme inhibition. Ultrafiltration separation of the peptides yielded fractions with stronger inhibitory activity against chymotrypsin while the effect on trypsin inhibition was dependent on type of enzyme used for protein hydrolysis. Future work should also examine the >10 kDa membrane fraction with respect to its in vitro inhibitory properties against trypsin and chymotrypsin. Inhibition of trypsin and chymotrypsin activities was revealed to be due to competitive binding of the peptides to the enzyme active site, which suggest ability of the peptides to fit into the active sites or interact with active site amino acid residues. Results from this work suggest that the enzymatic pea protein hydrolysates could function to improve human health by modulating metabolic or viral reactions that are dependent on serine protease activities. However, since the hydrolysates could be susceptible to proteolysis in the gastrointestinal tract, animal and human studies will be required to confirm health benefits. However, the hydrolysates may be useful for food preservation by preventing unwanted proteolysis or through toxic effects on pests that are dependent on serine proteases for survival. 

## Figures and Tables

**Table 1:** Enzyme hydrolysis conditions.

Enzyme	pH	Temperature (°C)
Alcalase	8.0	55
Chymotrypsin	8.0	37
Pepsin	2.0	37
Trypsin	8.0	37

**Table 2:** Kinetics constants for trypsin inhibition by protein hydrolysate and peptide fraction.

Catalytic		Trypsin Hydrolysate (TH)		<1 kDa Fraction of TH	
parameter	Control	0.25 mg/mL	5.0 mg/mL	0.25 mg/mL	5.0 mg/mL
Vmax (ΔAbs/min)	0.08 ± 0.01	0.08 ± 0.01	0.08 ± 0.01	0.08 ± 0.01	0.08 ± 0.01
Km (mM)	0.46 ± 0.04	0.91 ± 0.26	0.77 ± 0.26	0.55 ± 0.09	0.70 ± 0.13
Ki (mg/mL)		1.172 ± 0.28		1.073 ± 0.19	

**Table 3:** Kinetics constants for chymotrypsin inhibition by protein hydrolysate and peptide fraction.

Catalytic		Chymotrypsin Hydrolysate (CH)		1–3 kDa Fraction of CH	
parameter	Control	3.0 mg/mL	4.0 mg/mL	3.0 mg/mL	4.0 mg/mL
Vmax (ΔAbs/min)	0.019 ± 0.001	0.019 ± 0.001	0.019 ± 0.002	0.019 ± 0.001	0.019 ± 0.001
Km (mM)	0.011 ± 0.001	0.027 ± 0.004	0.078 ± 0.001	0.018 ± 0.004	0.025 ± 0.005
Ki (mg/mL)		0.763 ± 0.100		1.897 ± 0.900	

## References

[1] Kunitz M. (1945). Crystallization of a trypsin inhibitor from soybean. Science.

[2] Birk Y. (1985). The Bowman-Birk inhibitor. Trypsin and chymotrypsin inhibitor from soybeans. Int. J. Pept. Protein Res..

[3] Jin C.-Y., Zeng F.-K., Liu G. (2018). Recovery of protease inhibitors from potato fruit water by expanded bed adsorption chromatography in pilot scale. Am. J. Potato Res..

[4] Kostekli M., Karakaya S. (2017). Protease inhibitors in various flours and breads: Effect of fermentation, baking, and in vitro digestion on trypsin and chymotrypsin activities. Food Chem..

[5] Jamal F., Pandey P.K., Singh D., Khan M.Y. (2013). Serine protease inhibitors in plants: Nature’s arsenal crafted for insect predators. Phytochem. Rev..

[6] Quan T.H., Benjakul S. (2019). Trypsin inhibitor from duck albumen: Purification and characterization. J. Food Biochem..

[7] Klomklao S., Benjakul S., Simpson B.K. (2015). Inhibition of bigeye snapper (*Priacanthus macracanthus*) proteins by trypsin inhibitor from yellowfin tuna (*Thunnus albacores*) roe. J. Food Biochem..

[8] Choi J.-H., Park P.-J., Kim S.-K. (2002). Purification and characterization of a trypsin inhibitor from the egg of skipjack tuna *Katsuwonus pelamis*. Fisheries Sci..

[9] Rawdkuen S., Benjakul S., Visessanguan W., Lanier T.C. (2005). Fractionation and characterization of cysteine proteinase inhibitor from chicken plasma. J. Food Biochem..

[10] Harish B.S., Uppuluri K.B. (2018). Microbial serine protease inhibitors and their therapeutic applications. Int. J. Biol. Macromol..

[11] Sabotic J., Kos J. (2012). Microbial and fungal protease inhibitors-current and potential applications. Appl. Microbiol. Biotechnol..

[12] Shamsi T.N., Parveen R., Fatima S. (2016). Characterization, biomedical and agricultural applications of protease inhibitors. Int. J. Biol. Macromol..

[13] Rakashanda S., Qazi A.K., Majeed R., Andrabi S.M., Hamid A., Sharma P.R., Amin S. (2015). Plant-derived protease inhibitors LC-pi (*Lavatera cashmeriana*) inhibit human lung cancer cell proliferation in vitro. Nutr. Cancer.

[14] Al-Awadhi F.H., Law B.K., Paul V.J., Luesch H. (2017). Grassystatins D-F, potent aspartic protease inhibitors from marine cyanobacteria as potential antimetastatic agents targeting invasive breast cancer. J. Nat. Prod..

[15] Safavi F., Rostami A. (2012). Role of serine proteases in inflammation: Bowman-Birk protease inhibitor (BBI) as a potential therapy for autoimmune diseases. Exp. Mol. Pathol..

[16] Kennedy A.R. (1998). The Bowman-Birk inhibitor from soybeans as an anticarcinogenic agent. Am. J. Clin. Nutr..

[17] Lima A.I.G., Mota J., Monteiro S.A.V.S., Ferreira R.M.S.B. (2016). Legume seeds and colorectal cancer revisited: Protease inhibitors reduce MM-9 activity and colon cancer cell migration. Food Chem..

[18] Sun Y., Liu L., Zhang G.-F., Li G.-M., Wu N. (2013). Preparation, identification, structure, and in vitro anti-obesity effects of protease inhibitors isolated from potato fruit juice. Eur. Food Res. Technol..

[19] Ashton-Rickardt P.G. (2013). An emerging role for serine protease inhibitors in T lymphocyte immunity and beyond. Immunol. Lett..

[20] Jin T., Yu H., Wang D., Zhang H., Zhang B., Quezada H.C., Zhu W. (2016). Bowman-Birk inhibitor concentrate suppresses experimental autoimmune neuritis via shifting macrophages from M1 to M2 subtype. Immunol. Lett..

[21] Olanca B., Ozay D.S. (2015). Effects of natural protease inhibitors on high protease activity flours. J. Cereal Sci..

[22] Bijina B., Chellappan S., Krishna J.G., Basheer S.M., Elyas K.K., Bahkali A.H., Chandrasekaran M. (2011). Protease inhibitor from Moringa oleifera with potential for use as therapeutic drug and as seafood preservative. Saudi J. Biol. Sci..

[23] Klomklao S., Benjakul S., Kishimura H., Osako K., Simpson B.K. (2016). Purification and characterization of trypsin inhibitor from yellow tuna (*Thunnus albacores*) roe. J. Food Biochem..

[24] Boudida Y., Gagaoua M., Becila S., Picard B., Boudjellal A., Herrera-Mendez C.H., Sentandreu M., Ouali A. (2016). Serine protease inhibitors as good predictors of meat tenderness: Which are they and what are their functions?. Crit. Rev. Food Sci. Nutr..

[25] Singh A., Benjakul S. (2018). Proteolysis and its control using protease inhibitors in fish and fish products: A review. Comp. Rev. Food Sci. Food Saf..

[26] Singh A., Benjakul S. (2017). Serine protease inhibitors from squid ovary: Extraction and its effect on proteolysis and gel properties of surimi. J. Food Sci. Technol..

[27] Abd El-Latif O.A. (2015). Biopotency of serine protease inhibitors from cowpea (*Vigna unguiculata*) seeds on digestive proteases and the development of *Spodoptera littoralis* (Boisduval). Arch. Insect Biochem. Physiol..

[28] Bray G.A., Smith S.R., de Jonge L., Xie H., Rood J., Martin C.K., Redman L.M. (2012). Effect of dietary protein content on weight gain, energy expenditure, and body composition during overeating: A randomized controlled trial. J. Am. Med. Assoc..

[29] Wu G. (2013). Amino Acids: Biochemistry and Nutrition.

[30] Wu G. (2009). Amino acids: Metabolism, functions, and nutrition. Amino Acids.

[31] Lunagariya N.A., Patel N.K., Jagtap S.C., Bhutani K.K. (2014). Inhibitors of pancreatic lipase: State of the art and clinical perspectives. EXCLI J..

[32] Patil P., Mandal S., Tomar S.K., Anand S. (2015). Food protein-derived bioactive peptides in management of type 2 diabetes. Eur. J. Nutr..

[33] Aluko R.E. (2015). Antihypertensive peptides from food proteins. Annu. Rev. Food Sci. Technol..

[34] Udenigwe C.C., Aluko R.E. (2012). Food protein-derived bioactive peptides: Production, processing and potential health benefits. J. Food Sci..

[35] Fernandes J.P.C., Mehdad A., Valadares N.F., Mourão C.B.F., Ventura M.M., Barbosa J.A.R.G., de Freitas S.M. (2019). Crystallographic structure of a complex between trypsin and a nonapeptide derived from a Bowman-Birk inhibitor found in *Vigna unguiculata* seeds. Arch. Biochem. Biophys..

[36] Aguirrezabala-Cámpano M., Torres-Acosta R., Blanco-Labra A., Mediola-Olaya M., Sinagawa-García S., Gutiérrez-Díez A., Torres-Castillo J. (2013). Trypsin inhibitors in xoconostle seeds (*Opuntia joconostle* Weber.). J. Plant Biochem. Biotechnol..

[37] Awosika T.O., Aluko R.E. (2019). Inhibition of the in vitro activities of α-amylase, α-glucosidase and pancreatic lipase by yellow field pea (*Pisum Sativum* L.) protein hydrolysates. Int. J. Food Sci. Technol..

[38] Alashi A.M., Blanchard C.L., Mailer R.J., Agboola S.O., Mawson A.J., He R., Malomo S.A., Girgih A.T., Aluko R.E. (2014). Blood pressure lowering effects of Australian canola protein hydrolysates in spontaneously hypertensive rats. Food Res. Int..

[39] Markwell M.A.K., Haas S.M., Bieber L.L., Tolbert N.E. (1978). A modification of the Lowry procedure to simplify protein determination in membrane and lipoprotein samples. Anal. Biochem..

[40] Souza P.F.N., Vasconcelos I.M., Silva F.D.A., Moreno F.B., Monteiro-Moreira A.C.O., Alencar L.M.R., Oliveira J.T.A. (2016). A 2S Albumin from the seed cake of *Ricinus communis* inhibits trypsin and has strong antibacterial activity against human pathogenic bacteria. J. Nat. Prod..

[41] Malomo S.A., Aluko R.E. (2016). In vitro acetylcholinesterase inhibitory properties of enzymatic hemp seed protein hydrolysates. J. Am. Oil Chem. Soc..

[42] He R., Malomo S.A., Alashi A., Girgih A.T., Ju X., Aluko R.E. (2013). Purification and hypotensive activity of rapeseed protein-derived renin and angiotensin converting enzyme inhibitory peptides. J. Funct. Foods.

[43] Osman A., Goda H.A., Abdel-Hamid M., Badran S.M., Otte J. (2016). Antibacterial peptides generated by alcalase hydrolysis of goat whey. LWT Food Sci. Technol..

[44] Segura Campos M.R., Peralta González F., Chel Guerrero L., Betancur Ancona D. (2013). Angiotensin I-converting enzyme inhibitory peptides of chia (*Salvia hispanica*) produced by enzymatic hydrolysis. Int. J. Food Sci..

[45] Humiski L., Aluko R.E. (2007). Physicochemical and bitterness properties of enzymatic pea protein hydrolysates. J. Food Sci..

[46] Girgih A.T., He R., Hasan F.M., Udenigwe C.C., Gill T.A., Aluko R.E. (2015). Evaluation of the in vitro antioxidant properties of a cod (*Gadus morhua*) protein hydrolysate and peptide fractions. Food Chem..

[47] Erickson R.H., Kim Y.S. (1990). Digestion and absorption of dietary protein. Annu. Rev. Med..

[48] Ngai P.H.K., Ng T.B. (2004). A napin- like polypeptide from dwarf Chinese white cabbage seeds with translation- inhibitory, trypsin- inhibitory, and antibacterial activities. Peptides.

[49] Kuhar K., Kansal R., Subrahmanyam B., Koundal K., Miglani K., Gupta V. (2013). A Bowman–Birk protease inhibitor with antifeedant and antifungal activity from Dolichos biflorus. Acta Physiol. Plant..

